# From the Microscope to the Genome: A New Era in the Molecular Genetics of Epidermolysis Bullosa

**DOI:** 10.3390/ijms27177591

**Published:** 2026-08-25

**Authors:** Maria Tsvetkova, Tsonka Dimitrova, Galina Yaneva

**Affiliations:** Department of Biology, Faculty of Pharmacy, Medical University of Varna, 9000 Varna, Bulgaria

**Keywords:** epidermolysis bullosa, next-generation sequencing, molecular genetics, gene therapy, exon skipping, CRISPR-Cas9

## Abstract

Epidermolysis bullosa (EB) is a heterogeneous group of inherited disorders characterised by skin fragility, caused by pathogenic variants in genes encoding structural components of the dermo-epidermal junction. With the advent of next-generation sequencing (NGS), the diagnostic paradigm has shifted from a morphological to a genotype-oriented approach. This review summarises the genetic architecture of EB, the types of mutations and genotype–phenotype relationships, the challenges in interpreting variants of unknown significance (VUS), and therapeutic strategies targeting specific mutational mechanisms, including read-through approaches, exon skipping and genome editing. The role of modifier genes and epigenetic factors in clinical variability is also discussed. The focus is on the translational potential of genomics for personalized therapy in EB. Overall, this review synthesizes the molecular basis of all four major EB types across 16+ classical genes, highlights the paradigm shift where NGS achieves a diagnostic yield exceeding 90%, and critically assesses recent therapeutic milestones—ranging from the first FDA-approved topical gene therapy to precision RNA and genome-editing modalities.

## 1. Introduction

The end of diagnostic uncertainty: deciphering biological ‘fragility’.

In the history of dermatology, few conditions are as visually striking and, at the same time, as molecularly complex as epidermolysis bullosa (EB) [1,2]. For a long time, diagnosis was based on what the eye could see—the blister level and the severity of the clinical presentation [2,3]. Today, however, EB is no longer viewed simply as a skin disease, but as a defect in the molecular blueprint that governs skin integrity [1,4].

With the advent of next-generation sequencing (NGS), the question facing the clinician is no longer simply ‘What is the type of EB?’, but ‘Where exactly is the error in the code?’ or ‘Which specific nucleotide has been altered, and how does this change affect the thermodynamics of the dermo-epidermal junction?’ [5,6]. This shift from morphology to digital genetics not only shortens patients’ ‘diagnostic odyssey’, but also lays the foundations for an era in which diagnosis serves as a direct bridge to personalized therapy [5,7,8]. The primary objective of this review is to provide a comprehensive, state-of-the-art synthesis of the evolving molecular landscape of epidermolysis bullosa. Specifically, we aim to: (1) delineate the genetic architecture and basement membrane protein interactions underlying EB pathogenesis; (2) evaluate the clinical transition from ultrastructural diagnostics to next-generation genomic methodologies; (3) review emerging molecular and cellular therapies; and (4) discuss current translational hurdles, non-invasive surveillance tools, and future directions toward personalized genomic medicine.

## 2. Architecture of Resilience: The Skin as a Molecular Construct

**Figure 1:**
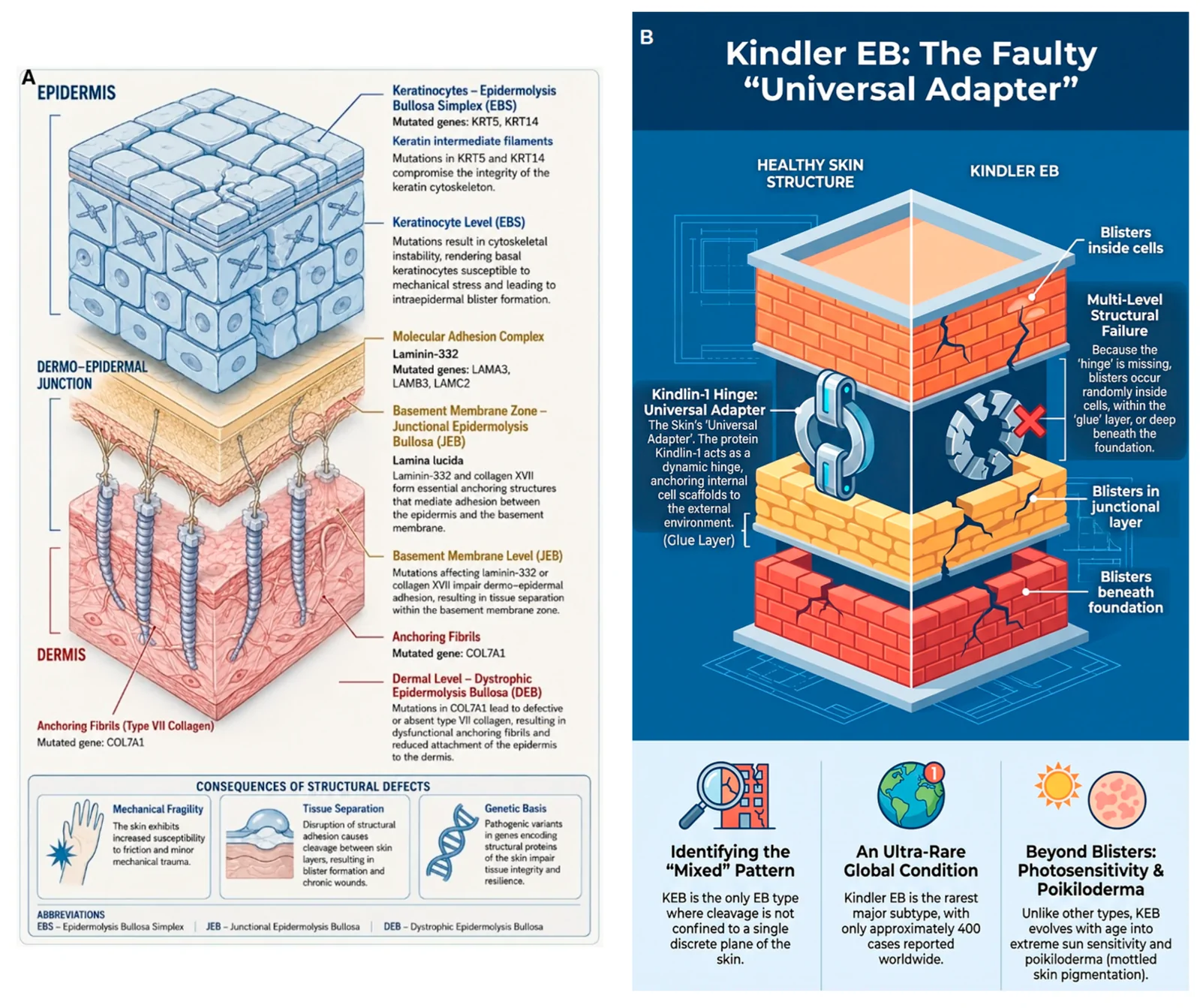
Structural architecture and plane separation patterns in Epidermolysis Bullosa (EB). (**A**) Schematic representation of skin architecture and plane separation across classical EB subtypes: intraepidermal cleavage in Epidermolysis Bullosa Simplex (*KRT5, KRT14*), intra-lamina lucida separation in Junctional Epidermolysis Bullosa (*LAMA3, LAMB3, LAMC2, COL17A1*), and sub-lamina densa cleavage in Dystrophic Epidermolysis Bullosa (*COL7A1*). (**B**) Architectural collapse and molecular pathogenesis in Kindler Epidermolysis Bullosa (KEB), illustrating mixed levels of plane cleavage (intraepidermal, junctional, and dermal) resulting from Kindlin-1 (*FERMT1*) deficiency (the red cross (x) denotes the absence/loss of functional Kindlin-1 adapter protein). Colors represent distinct anatomical skin layers and cleavage planes: blue indicates the epidermis/keratinocyte layer, yellow/gold denotes the dermo-epidermal junction/basement membrane zone, and red/pink corresponds to the dermis/sub-lamina densa zone. Created by the authors.

To date, pathogenic variants have been identified in 16 different genes that encode proteins responsible for the structural integrity of keratinocytes and the dermo-epidermal junction [1,2,5]. The condition is classified into four main types according to the level of tissue separation [1,2,3] (Table 1):

To understand the genetics of EB, we must view the skin not as a mere covering, but as a complex engineered system of ‘anchors’ (clips) and ‘rivets’, where, if a single clip is missing, the entire structure (in this case, the skin) collapses. The formation of blisters in patients is due precisely to genetically determined defects in the structures of the epidermis, the dermo-epidermal junction and the dermis [1,2]. Depending on the exact level of this ‘engineering structure’ at which the problem arises, the condition is classified accordingly (Figure 1A,B):

‘Keratinocyte’ level (EB Simplex): The condition affects the outermost layer of the skin, the epidermis, and more specifically its lowest, basal layer. Here, the problem lies in the intracellular cytoskeleton of the “bricks” themselves (basal keratinocytes) [9,10]. Genetic defects in the primary structural filaments, keratin 5 (*KRT5* gene) and keratin 14 (*KRT14* gene), cause the keratin filaments to collapse. When cells cannot maintain their shape, it disintegrates under the slightest pressure (basal cytolysis), leading to the formation of intraepidermal blisters [1,2] (Figure 1A).

‘Junctional EB’ level: This is the molecular ‘glue’ of the lamina lucida (the dermo-epidermal junction). Defects in laminin-332 (encoded by the *LAMA3, LAMB3, LAMC2*) disrupt the connection between the epidermis and the dermis [1,2,11]. Type XVII collagen (*COL17A1* gene) is a transmembrane protein that spans the cell membrane and acts as a structural bridge connecting the interior of the cell to the lamina densa [1,9]. This is the most critical zone, where even a small change in protein configuration can be fatal (Figure 1A).

‘Foundation’ level (Dystrophic EB): This is located beneath the dermo-epidermal junction (sublamina densa) and serves as the scaffold that anchors the entire skin structure to the deep tissues of the body. The *COL7A1* gene, which encodes type VII collagen, plays a key role here [11]. As this collagen forms the ‘anchoring fibrils’ that literally attach the dermis to the epidermis, its absence leads to extremely fragile skin that tears at the slightest touch, causing scarring (dystrophy) and permanent tissue loss [1,2,12] (Figure 1A).

“Mixed” Level (Kindler EB)—“Universal Adapters”: Here, the engineering element is the protein kindlin-1 (*FERMT1* gene), which forms the cell’s focal adhesions [2]. In our analogy, these are the universal adapters or “dynamic hinges” that connect the internal scaffold of the “bricks” (cells) to the external environment [1,2]. When these connecting adapters are missing or defective, structural instability is not confined to just one specific “floor” of the building. Cracks and blisters appear at mixed and random levels within the structure—the rupture can occur both inside the cells themselves (as in EBS) and in the “glue” (as in JEB), or deep beneath the “foundation” (as in DEB) [2] (Figure 1B).

## 3. The Diagnostic Turning Point: The Collapse of Invasiveness

For decades, the diagnostic pathway for patients with epidermolysis bullosa (EB) began with an injury. To determine the subtype of the condition, doctors had to perform a skin biopsy, as the traditional ‘gold standard’—namely, immunofluorescence mapping (IFM) (using antibodies against specific proteins) and electron microscopy—required a fresh skin biopsy from a newly formed blister [2,3]. In newborns with skin as delicate as a ‘butterfly’s wing’, this is not only painful but also risky, as the procedure itself causes new wounds and carries a risk of infection.

Today, the modern strategy is shifting towards the so-called ‘genetics-first’ approach—next-generation sequencing (NGS)—which has completely ‘dethroned’ the old invasive approach, becoming the new ‘gold standard’ [5,6].

### 3.1. NGS—The New ‘Gold Standard’

The introduction of NGS addresses key diagnostic challenges in three areas:

- Pain-free diagnosis (non-invasiveness): ‘The power’ of a single drop

The greatest revolution brought about by NGS is the possibility of non-invasive sampling. Instead of a scalpel and a biopsy needle, the modern geneticist uses an oral swab (buccal swab) or saliva, or a drop of blood: a minimal amount, sufficient to isolate genomic DNA [5,6].

For the newborn and their parents, this means sparing them unnecessary pain in the first days of life. NGS allows the simultaneous analysis of all 16+ known genes for BE in a single test (Targeted Gene Panels). This eliminates ‘diagnostic guesswork’ and provides a definitive answer within weeks rather than months [5,6].

- Higher diagnostic accuracy: Detection of mosaicism (where only some of the cells carry the mutation), which is often missed by microscopy; detection of atypical phenotypes via Whole Exome Sequencing (WES), where standard panels fail to detect a mutation; and detection of mutations in non-coding (intron) regions via Whole Genome Sequencing (WGS), which are often missed by WES [5,6].
- Prognostic value: It is now possible to predict the risk of squamous cell carcinoma (cSCC), which occurs in dystrophic BE, even before the first clinical signs appear, based on the specific domain in which the mutation is located—methods for non-invasive monitoring through the analysis of circulating tumour DNA and specific proteins in serum, which could predict the severity of the disease even at an early age [5,7].

This is of paramount importance, not only because rapid and accurate genetic diagnosis is critical for both prognosis and prenatal diagnosis, as well as for planning modern therapy [7,8], but also because there is now a humanitarian dimension to technological progress. We are talking here not just about better machines, but about putting an end to the physical suffering involved in the diagnostic process for these vulnerable patients.

### 3.2. The ‘VUS’ Challenge: The Genetic Maze

Despite its immense power, NGS presents specialists with a new dilemma, namely variants of unknown significance (VUS). Sequencing often reveals changes in the genetic code (mutations) that have not yet been described in the scientific literature, and there is already talk of the so-called ‘grey area’ of diagnostics. In such cases, the geneticist cannot immediately provide a definitive prognosis on whether the variant is a being polymorphism or a pathogenic mutation [5,6]. This necessitates additional family studies (sequencing of the parents) or functional analyses, which are discussed in the following sections [6]. Standardizing the interpretation of Variants of Uncertain Significance (VUS) across international diagnostic centers requires a multi-tiered approach. Implementing disease-specific ACMG/AMP (American College of Medical Genetics and Genomics/Association for Molecular Pathology) guidelines [13], leveraging curated databases (such as ClinGen EB Expert Panels) [14], and integrating functional assays—particularly transcriptomic profiling (RNA-seq) to detect aberrant splicing—are critical steps to reduce diagnostic ambiguity and facilitate accurate genotype–phenotype reclassification.

### 3.3. The New Role of Biopsy

The introduction of the new gold standard, however, does not completely rule out biopsy; rather, its role has now been transformed. It is no longer the primary means of diagnosis, but rather a tool for confirmation. If NGS detects the aforementioned VUS, doctors can revert to immunofluorescence mapping on a skin sample to see whether the relevant protein (e.g., collagen VII) is actually absent from the tissue [2,3].

The transition to NGS is not merely a technical change in method, but an ethical choice for more compassionate medicine. Diagnostics is shifting from the operating theatre to the bioinformatics laboratory.

## 4. Therapy-Oriented Diagnostics: When the Mutation Dictates the Treatment

Until recently, genetic diagnosis of EB was more of an academic exercise with little practical benefit for day-to-day treatment. Today, however, the genetic report forms the basis of the treatment protocol [7,8,15]. It is no longer ‘epidermolysis bullosa’ that is treated, but rather the specific molecular defect (Table 2).

### 4.1. The ‘Read-Through’ Strategy: Correcting Punctuation

About 15–20% of patients with severe forms of EB have so-called nonsense mutations (premature stop codons). Imagine that a cell is reading the genetic instructions for building collagen, but there’s a period in the middle of the sentence. The result is a nonfunctional, “truncated” protein. Modern diagnostic panels identify these specific stop codons, making patients candidates for small-molecule “read-through” therapy [15]. These therapies “trick” the cell into ignoring the erroneous stop codon, inserting a semantically similar amino acid, and completing the synthesis of the full-length protein, while simultaneously blocking the cellular mechanism for degrading defective mRNAs (nonsense-mediated decay (NMD)) [1,8].

- Gentamicin (an aminoglycoside) is an inexpensive and widely available antibiotic that has been shown to restore the expression of collagen VII in Recessive Dystrophic Epidermolysis Bullosa (RDEB) and laminin-332 (*LAMB3* gene) in JEB [1,7,8]. Its topical application (in the form of a 0.3% eye ointment) rapidly restores integrity and heals erosions in patients with JEB. Systemic intravenous administration (tested in Phase I/II clinical trials) also leads to synthesis of new, functional collagen fibrils and alleviates disease severity for months [8], but its long-term use is limited due to the cumulative risk of nephrotoxicity and ototoxicity. This makes local delivery a safer and preferred method. To optimize treatment, combinations of low doses of gentamicin, paromomycin, and antioxidants are also being developed [7].
- Ataluren (PTC124), target small molecule, is a specialized agent designed to overcome pathogenic premature stop codon; a major advantage is that it does not affect the natural (normal) stop codons at the end of genes. Although it was initially developed for muscular dystrophy, it has demonstrated clinical efficacy in alleviating the severity of lesion and complications in patients with JEB [7].
- Amlexanox is an anti-inflammatory drug that potently inhibits the NMD pathway and induces a read-through effect. When applied to RDEB cells in vitro, it generates full-length collagen VII. Its main advantage over aminoglycosides is its significantly more favorable toxicity profile [8].
- Atazanavir and Artesunate directly target the ribosomal protein L35, promoting read-through specifically of *LAMB3* transcripts without disrupting global translation—an extremely promising approach for newborns with severe JEB [8].

While nonsense mutation read-through strategies (e.g., aminoglycosides such as gentamicin) can restore full-length functional laminin and collagen proteins, systemic administration carries significant risks of cumulative nephrotoxicity and ototoxicity. Consequently, localized topical formulations or next-generation, non-aminoglycoside read-through compounds (such as ataluren/PTC124 and synthetic designer macrolides) represent a safer therapeutic window for long-term clinical management [7,8].

### 4.2. RNA-Based Therapies: ASOs or the “Art” of Exon Skipping and SMaRT

Unlike DNA editing, which aims to permanently alter the genome, therapies targeting RNA or the translational machinery offer temporary but potentially safer modulation of gene expression. Since they do not permanently alter DNA, the risk of oncogenesis or unwanted genomic mutations is minimized, although they require lifelong administration [16].

- Antisense oligonucleotides (ASOs) are short single-stranded molecules (typically 15–25 nucleotides) that bind specifically to the target pre-messenger RNA (pre-mRNA) and modulate its splicing without altering genomic DNA [11]. Their most established application in EB is the exon-skipping strategy, in which the ASO molecule “masks” the mutated exon, rendering it invisible to the cellular splicing machinery [11]. As a result, the defective exon is spliced out along with the introns, restoring the open reading frame. This results in the synthesis of a truncated but partially functional protein, which can mitigate the phenotype from severe to milder [8,11].

The application of this strategy to *COL7A1* (dystrophic EB) is particularly suitable, as over 90% of the exons in the *COL7A1* gene are in-frame (they maintain the reading frame upon removal) [11]. Preclinical studies have successfully demonstrated the skipping of exons 13, 70, 73, 80, and 105 [8,14]. The greatest clinical progress has been achieved with QR-313—an antisense oligonucleotide (ASO) developed to induce exon 73 skipping in the *COL7A1* gene. Formulated as a topical carbomer hydrogel for direct application to wounds, QR-313 has shown promising results in preclinical studies, restoring type collagen VII expression and improving dermo-epidermal adhesion. However, the Phase I/II clinical trial demonstrated limited uptake and efficacy, which has spurred the development of next-generation ASO candidates, such as PTW-002 [7,8,11,16]. In other studies, ASO molecules targeting exon 80 achieved up to 90% exon skipping efficiency in fibroblasts and restored levels of key proteins [11].

Regarding the application of the exon-skipping strategy in JEB, the *COL17A1* gene is also an excellent target for ASOs, as exon 6 and exon 52 skipping have been successfully demonstrated for this gene [16,17]. For *LAMB3*, however, there are no data in the available literature on specific ASO-mediated exon skipping. The reason is that mutations in *LAMB3* (encoding laminin-332) often require the full length of the protein, which is why gene replacement therapies or read-through approaches are preferred for this gene [8].

- Spliceosome-mediated RNA trans-splicing (SMaRT) is another type of innovative RNA-based technology that utilizes the endogenous cellular spliceosome, to replace large mutated regions of the patient’s own pre-mRNA with healthy RNA sequences provided by an exogenous molecule called an RNA trans-splicing molecule (RTM) [8,16]. It enables the correction of multiple different mutations in a given gene with a single product [8]. In preclinical in vitro and in vivo models, SMaRT has successfully corrected mutations in the *PLEC* and *KRT14* genes (in EBS), as well as *COL7A1* (in DEB) and *COL17A1* (in JEB) [8,16]. Despite its enormous potential, the technology is hampered by several factors, such as the relatively low efficiency of trans-splicing compared to natural (cis) splicing, the risk of off-target effects, and the difficulties in delivering large RTMs through the skin barrier [8]. Furthermore, similar to ASOs, the effect is transient and requires lifelong treatment [16]. Spliceosome-Mediated RNA Trans-Splicing (SMaRT) offers the distinct advantage of repairing large mutational hotspots across entire gene regions using a single pre-trans-splicing molecule. However, compared to antisense oligonucleotides (ASOs) that mediate targeted exon skipping, SMaRT currently exhibits lower basal trans-splicing efficiency, experiences strong competition with endogenous cis-splicing machinery, and requires complex delivery constructs [8,16].

### 4.3. In Vivo Gene Delivery Therapy: Topically Administered Viral Vectors (B-VEC)

The Food and Drug Administration (FDA)-approved therapy Beremagene geperpavec (B-VEC) uses a non-replicating herpes simplex virus type 1 (HSV-1) vector to deliver two functional copies of the *COL7A1* gene directly into wounds. Since HSV-1 does not integrate into the host genome, B-VEC acts as an episomal system, restoring collagen VII without the risk of insertion mutagenesis [15,18].

### 4.4. Precision Genome Editing (CRISPR, Base, and Prime Editing): Beyond “Copy-Paste”

The most exciting prospect in current reviews is the shift from adding genes to editing them directly in situ. With CRISPR/Cas9, Base and Prime Editing, the aim is no longer simply to introduce a new gene via a virus (as is the case with the approved B-VEC therapy), but rather to ‘delete’ the mutation and restore the original sequence in the patient’s genome [9,10,17,19,20,21]. Despite promising preclinical results, clinical translation of in vivo CRISPR/Cas9 editing faces key limitations, including potential off-target double-strand breaks, vector immunogenicity, and low homology-directed repair (HDR) efficiency in post-mitotic cutaneous tissues. Overcoming the physical stratum corneum barrier without inducing shear-stress blistering in fragile skin necessitates optimized non-integrating viral platforms (e.g., topical HSV-1 vectors), deformable liposomes, lipid nanoparticles (LNPs), or biocompatible dissolvable microneedle arrays [17,19]. The future of EB diagnostics will involve a ‘safety scan’ of the entire genome before commencing a CRISPR procedure.

All this shows that DNA is not the only factor determining a patient’s outcome.

**Table 2:** Genotype–therapy associations in EB.

Gene	Target Mutation	EB Subtype	Therapeutic Approach	Source
*COL7A1*	Nonsense (PTCs)	RDEB	Read-through: Gentamicin	[1,7,8,15]
*COL7A1*	Nonsense (PTCs)	RDEB	Read-through: Amlexanox	[8]
*COL7A1*	Exon 73 mutations	DEB/RDEB	Exon skipping (ASO): QR-313 (Hydrogel)	[7,8,11]
*COL7A1*	Hotspot exons (13, 70, 80, 105)	DEB/RDEB	Exon skipping (ASO)	[8,11,16]
*COL7A1*	Null/Any pathogenic variant	RDEB/DDEB	In vivo Gene Addition: B-VEC/Vyjuvek (HSV-1)	[15,18]
*COL7A1*	Frameshift/Point variants	RDEB	Genome Editing: CRISPR-Cas9/Base/Prime Editing	[9,10,17,19,20,21]
*LAMB3*	Nonsense (PTCs)	JEB	Read-through: Ataluren/PTC124	[7]
*LAMB3*	PTCs (severe neonatal)	JEB	Read-through: Atazanavir/Artesunate	[8]
*COL17A1*	Recessive splice site/Exons 5–7	JEB	Exon skipping: Targeted ASOs	[16,17]
*KRT5*	Dominant-negative (Exon 1)	EBS	RNA Trans-splicing: Synthetic PTMs	[8,16]
*KRT14*	Dominant-negative missense	EBS (Dowling-Meara type)	Allele-Specific Silencing: CRISPR-Cas9; RNA trans-splicing (SMaRT)	[8,10,20]
*PLEC*	Recessive truncating/Splice site	EBS (EBS-MD and EBS-PA)	Gene Addition/Prime Editing—CRISPR/Cas9; RNA Trans-Splicing (SMaRT); Natural Exon Skipping	[1,8,10,11,15,16]

## 5. The Regenerative Revolution: From Controlling Fibrosis to Growing Healthy Grafts

In the search for long-lasting and effective solutions for Epidermolysis Bullosa (EB), the focus of scientific research is increasingly expanding beyond classical gene therapy to encompass innovative approaches in regenerative medicine. These strategies aim not only to restore the missing protein but also to modulate the tissue microenvironment, suppress inflammation, and create fully functional skin substitutes [7].

### 5.1. Use of Mesenchymal Stem Cells (MSCs) to Reduce Inflammation and Fibrosis

Mesenchymal stem cells (MSCs) possess unique potential for self-renewal and differentiation, but their primary therapeutic value in EB stems from their potent immunomodulatory and anti-inflammatory properties [7]. In patients with RDEB, chronic tissue damage leads to severe inflammation and subsequent fibrosis. The administration of MSCs significantly reduces tissue inflammation, which is a key mechanism for alleviating the fibrotic aspects of wounds and promoting healing [12]. Clinical trials use allogeneic (donor) MSCs derived from various sources, including the umbilical cord, Umbilical Cord tissue-derived Mesenchymal Stem Cells (UC tissue-MSCs), for intravenous infusions and adipose tissue (Adipose-derived Stem Cells—ASC), from which cell sheets are generated for local application to wounds [8]. Since allogeneic MSCs are characterized by low immunogenicity, they can be safely administered (systemically or topically) without the need for aggressive immunosuppression regimens in the recipient [7].

### 5.2. Tissue Engineering—3D Bioprinted Skin Equivalents

Tissue engineering offers the possibility of creating three-dimensional (3D) skin equivalents that structurally and morphologically resemble the architecture of human skin. These substitutes are constructed by culturing keratinocytes and fibroblasts on specialized matrices, such as decellularized human dermal matrix [20]. Innovative therapeutic strategies for direct wound coverage are also being developed, such as a pilot clinical trial evaluating a wearable device for applying a non-invasive, electro-spun nanofibrous matrix (Spincare^®^ Matrix, Nanomedic Technologies Ltd., Lod, Israel) directly onto lesions in RDEB patients to stimulate healing [7]. In addition to serving as temporary wound dressings, 3D skin equivalents act as an ideal “scaffold” for transplanting gene-edited cells back into the patient, ensuring proper protein localization at the dermo-epidermal junction [8,16].

### 5.3. Induced Pluripotent Stem Cells (iPSCs) and CRISPR for Autologous Healthy Grafts

One of the major limitations of classical ex vivo cell therapies is the short lifespan of primary skin cells and the need for large amounts of material. This problem is solved by induced pluripotent stem cells (iPSCs), which possess unlimited proliferative potential and can differentiate into the desired skin cell types—keratinocytes and fibroblasts [7,9,16]. By combining iPSCs with the precision of CRISPR/Cas9 gene editing, scientists can correct pathogenic mutations (e.g., in the *COL7A1* gene) directly in cells extracted from the patient [7,16,22,23]. Following molecular correction, these healthy iPSCs are differentiated and used to generate autologous 3D skin composites (iSC grafts) [22]. The transplantation of such gene-edited skin equivalents demonstrates normal expression of the missing protein (collagen VII), restoration of anchoring fibrils, and formation of a stable stratified epidermis, offering a safer, long-lasting, and virus-free alternative for the permanent treatment of the most severe forms of EB [9,22,23].

## 6. When a Missing Foundation Leads to Cancer: Decoding Squamous Cell Carcinoma and the Hope of Immunotherapy

Cutaneous squamous cell carcinoma (cSCC) is the most serious and fatal complication in patients with severe recessive dystrophic epidermolysis bullosa (RDEB). It is characterized by early onset (in late adolescence or early adulthood) and an extremely aggressive metastatic potential, with cumulative mortality reaching 79% by age 55 [1,12].

### 6.1. Molecular Mechanisms and the Role of the Tumor Microenvironment

Unlike sporadic cSCC, the exceptional aggressiveness of cancer in RDEB is primarily due to the altered, tumor-promoting microenvironment, rather than a fundamentally different oncogenic profile [1]. The complete absence of collagen VII (C7) eliminates its tumor-suppressive effect and leads to hyperactivation of transforming growth factor-beta (TGF-β) signaling [1,12]. Elevated TGF-β stimulates the transdifferentiation of fibroblasts into cancer-associated myofibroblasts, which, through the secretion of lysyl oxidase, increase the stiffness of the extracellular matrix [1]. This stiffened microenvironment, combined with chronic bacterial colonization *(S. aureus*, *S. pyogenes*) and persistent inflammation, enhances the epithelial–mesenchymal transition (EMT), increases levels of matrix metalloproteinases (MMP-2), and suppresses local immune surveillance [1,12]. Genomically, this process is characterized by a mutational “signature” induced by apolipoprotein B mRNA-editing enzyme catalytic polypeptide-like (APOBEC) deaminases [1,24].

### 6.2. Targeted Therapies for RDEB-cSCC

The treatment of cSCC in patients with RDEB is extremely challenging. Wide local surgical excision remains the method of choice, but tumors often metastasize despite clear surgical margins [1]. Conventional radiation therapy and chemotherapy are generally ineffective and are used primarily for palliative purposes [1,9]. Consequently, the focus is shifting toward new biological and targeted approaches.

- One such approach is immunotherapy using immune checkpoint inhibitors (PD-1 inhibitors). Since systemic immunosuppression carries the risk of accelerating cancer progression in RDEB, the goal of modern therapy is to preserve and stimulate antitumor immunity [12]. Monoclonal antibodies against PD-1 (such as Nivolumab and Cemiplimab) are being studied in Phase I/II clinical trials specifically for patients with BE and advanced cSCC, showing promising results [8,9].
- Multikinase inhibitors (Rigosertib) act by inhibiting the enzyme Polo-like kinase 1 (PLK1). In preclinical models, Rigosertib has demonstrated the ability to induce apoptosis in RDEB cSCC keratinocytes in vitro and to reduce tumor growth in in vivo mouse xenografts [1]. Phase I/II clinical trials are currently underway to evaluate the objective therapeutic response to oral and intravenous administration of Rigosertib in patients with RDEB-cSCC [1,8].
- EGFR (Epidermal Growth Factor Receptor) inhibitors are also being considered as a potential biological therapy to limit tumor invasion, although clinical evidence of their efficacy in the specific context of RDEB is currently limited and requires further study [1].

Given the high propensity for aggressive cutaneous squamous cell carcinoma (cSCC) in patients with severe recessive dystrophic EB, non-invasive early surveillance tools are increasingly vital. Liquid biopsy platforms monitoring circulating tumor DNA (ctDNA) for hallmark somatic alterations (such as driver mutations in *TP53*, *HRAS*, and *NOTCH1*), coupled with wound microRNA profiles, offer promising potential for detecting occult malignant transformation prior to macroscopically visible ulceration [1,12].

## 7. Challenges: Beyond the Genetic Code

Even with whole-genome sequencing, not all questions can yet be answered. The molecular genetics of epidermolysis bullosa (EB) encounters phenomena that suggest DNA is only the beginning of the story.

### 7.1. Gene Modifiers and Epigenetics: The Paradox of the Identical Mutation

One of the greatest puzzles in modern genetics is the question of why two brothers carrying an absolutely identical pathogenic variant (mutation) can have dramatically different clinical presentations. One may suffer from extensive erosions and severe scarring, whilst the other leads a relatively normal life. The long-term changes observed in cell cultures of keratinocytes and fibroblasts from patients with RDEB suggest the presence of powerful epigenetic modifiers and persistent cellular responses to the microenvironment [12].

Factors such as DNA methylation or the activity of specific microRNAs (miRNAs) can ‘amplify’ or ‘attenuate’ the expression of the defective gene [2]. Although the specific role of these mechanisms in EB has yet to be fully demonstrated, persistent changes in the gene expression of key regulators such as decorin and transforming growth factor-beta (TGF-β) have already been identified in RDEB. These changes are both a secondary effect of chronic wound healing and the result of discrete epigenetic regulations; in both cases, they represent a key target for the development of new therapies aimed at controlling fibrosis [2,12]. The skin’s microenvironment, such as levels of inflammation, the bacterial microbiome and even vitamin D levels, act as biological ‘shock absorbers’ that determine how severely the genetic defect will manifest [12].

Phenotypic variability among patients with the same mutations (even within a single family) clearly demonstrates the presence of additional genetic modifiers [2,12]. It has been established that deep intronic recessive mutations in *COL7A1* can lead to marked intrafamilial phenotypic heterogeneity [8]. Furthermore, additional genetic variants in a cis position can also alter the expression of the corresponding allele by causing in-frame exon skipping of the exon containing the pathogenic variant. This significantly mitigates the severity of the disease, as the resulting truncated molecules often retain partial function [2].

There are ‘secondary’ genes which do not cause EB but influence how the skin responds to the defect. For example, the expression levels of the MMP-1 (Matrix Metalloproteinase-1) protein or the MMP1 gene can modulate the degree of fibrosis and wound healing [12]. In addition to this well-known single-nucleotide polymorphism (SNP) in the MMP-1 gene promoter, which leads to an imbalance between the synthesis and degradation of collagen VII in more severely affected patients [2,12], RDEB is also associated with pathological changes in the expression and activity of proteases such as MMP-2, MMP-3, MMP-7, MMP-9, MMP-13, and MMP-14 [12]. Studies of brothers with RDEB and a marked discrepancy in clinical severity show elevated levels of MMP-1, MMP-2, MMP-3, and MMP-9, with the moderately affected sibling showing the highest activity specifically for MMP-2 and MMP-9 [12].

Inflammatory cytokines (IL-6, TNF-α, and others) are another factor influencing disease severity. Chronic tissue damage and the associated impaired healing in EB lead to the chronic activation of inflammatory pathways. Serum samples from patients with dystrophic EB show high levels of inflammatory molecules such as interleukin-1β (IL-1β), IL-2, IL-6, IL-12, TNF-α, TNF-β, IFN-γ, and T-helper 17 cytokines, which promote scarring and fibrosis [8,25]. Interleukin-6 (IL-6) is of particular prognostic significance; lower levels of IL-6 (along with TGF-β) correlate with a milder course of the disease [12]. At the same time, the ratio of pro-inflammatory IL-6 to anti-inflammatory IL-10 in serum is considered a reliable prognostic marker for assessing the severity of RDEB [12].

Marked phenotypic discordance among patients or siblings harboring identical pathogenic mutations underscores the role of genetic and epigenetic modifiers. Secondary pathways—including polymorphisms in TGF-β1 signaling, matrix metalloproteinases (MMP-1, MMP-9), microRNA expression profiles, and differential promoter methylation—substantially modulate downstream fibrosis, chronic inflammation, and wound-healing dynamics [2,12].

### 7.2. Reversible Mosaicism as “Natural Gene Therapy”: When Nature Corrects Itself

The presence of somatic or germline mosaicism in epidermolysis bullosa poses significant diagnostic and therapeutic challenges [26,27]. With next-generation sequencing (NGS), standard approaches may miss low-frequency somatic mosaic variants (low-grade mosaicism) because they are present in only a small percentage of the cells in the sample [5,7].

In this context, the so-called phenomenon of “natural gene therapy” or the phenomenon of reversible mosaicism (RM) is particularly interesting. RM is caused by spontaneous secondary mutations that “correct” the original defect in a specific cell, which then proliferates [26,27,28]. Clinically, this manifests as patches of completely healthy skin observed in some patients amidst a sea of blisters [7,26]. This spontaneous correction most often occurs during mitosis through various genetic mechanisms, including true back mutation, intragenic crossover, mitotic gene conversion, or a compensatory second-site mutation [26]. The proliferation of these individual corrected cells into clearly visible healthy regions is explained by the “late-but-fitter” model, according to which revertant cells may arise at a later stage of development, but because they produce a healthy protein, they gain a selective advantage for survival and cell proliferation over the surrounding defective cells [26,28]. Furthermore, while it was long believed that RM occurred exclusively in epidermal keratinocytes, recent studies conclusively demonstrate that dermal fibroblasts also have the capacity to undergo genetic reversion. This is a key finding for RDEB, as it demonstrates that corrected fibroblasts are capable of independently restoring skin function and maintaining healthy tissue architecture in these patients [28].

From a diagnostic standpoint, detecting these complex recombinations in RM is difficult using standard Polymerase Chain Reaction (PCR) and cDNA analysis due to the risk of artificial recombination or mRNA degradation via nonsense-mediated decay (NMD). To precisely validate allele-specific expression, modern technologies such as Targeted RNA Sequencing (TREx) [28] and nanopore Cas9-targeted sequencing (nCATS) [27] are now being used. nCATS enables the sequencing of long DNA segments directly at the genomic level, avoiding the risk of artificial recombination that standard PCR can cause [27]. Given that cells with reversible mosaicism are naturally corrected, they represent a safe autologous platform for cell therapy. Their use for cultured epidermal autografts or for generating induced pluripotent stem cells (iPSCs) completely eliminates the risks of viral integration, immunogenicity, and off-target effects characteristic of artificial gene editing [1,7,26].

Understanding these mechanisms through spatial transcriptomics is a key challenge; if we understand how the skin heals itself, we will be able to mimic this process through medicine.

### 7.3. Variants of Unknown Significance (VUS) and the Role of Functional Genomics

VUS represent another major barrier today. With the advent of next-generation sequencing (NGS), the diagnosis of EB has undergone a revolution, but at the same time has faced a serious challenge—the dramatic increase in the number of Variants of Unknown Significance (VUS) detected. Data show that while VUS are detected in only 5% of cases with classical Sanger sequencing, this percentage jumps to 22% with NGS [5]. The discovery of this aforementioned “grey area” in diagnostics (VUS) creates a serious clinical dilemma and hinders the establishment of an accurate diagnosis and prognosis in real time [5,6].

To move beyond this “grey area,” bioinformatics analysis and clinical judgment alone are insufficient. This necessitates the urgent development of functional genomics—an approach in which the suspected defect is tested and validated in a laboratory setting [5]. The most advanced method for evaluating VUS involves creating 3D skin equivalents (organoids) from the patient’s own cells. Using these, scientists can directly observe whether a specific mutation leads to a lack of key structural proteins (e.g., collagen VII or laminin-332), whether it causes keratin filaments to collapse, or whether it disrupts the thermodynamics of the dermo-epidermal junction [23].

Combining functional in vitro assays with family-based genetic testing (sequencing of the parents) allows VUS to be correctly reclassified as pathogenic or benign. This step is absolutely essential because modern therapies (such as exon skipping or gene editing) are strictly mutation-specific and cannot be applied unless the pathogenicity of the variant has been conclusively proven [5,23].

### 7.4. Deep Intronic Variants and the Role of WGS and RNA-Seq

Although NGS (including targeted gene panels and whole-exome sequencing—WES) is the new gold standard, these approaches have their technological limitations [5,6]. Current WES and panel-based strategies primarily cover protein-coding exons and their immediate flanking regions (typically ±20 intronic nucleotides). Consequently, pathogenic variants in deep intronic regions, mutations in regulatory elements, and complex copy number variations are often missed, leading to genetically undiagnosed patients despite a definitive clinical presentation [5].

To address these gaps in clinical practice, whole-genome sequencing (WGS) is increasingly being used. WGS covers the entire genome and has the capacity to identify mutations in non-coding DNA regions, as well as complex chromosomal rearrangements that WES cannot detect [4,6].

Even when WGS detects a deep intronic variant, however, proving its pathogenicity is challenging. Here, too, RNA-transcriptome sequencing (RNA-seq) plays a critical role. It allows for functional validation of how a specific intron mutation or regulatory defect alters normal splicing (e.g., the creation of new cryptic splicing sites) and the final expression of messenger RNA [5].

In the future, the integration of WGS and RNA-seq, supported by advanced bioinformatics algorithms for data analysis, will be absolutely essential for identifying the missing genetic causes in unresolved cases of EB [5].

## 8. Global Inequality: The Cost of ‘Digital Healthcare’

A critical look at the future of EB would not be complete without considering the economic reality and global inequalities. While next-generation sequencing and personalized molecular therapies are a triumph of science, their clinical application currently remains a privilege reserved primarily for developed countries [1,7].

### 8.1. The Diagnostic Divide

Whilst NGS panels are part of the standard protocol in Europe and the United States, in many regions of Latin America, Africa and Asia, diagnoses are still made solely on the basis of clinical features [5,7]. This creates a critical barrier to precision medicine: new targeted therapies—such as the approved gene therapy B-VEC (Vyjuvek) or ASO molecules—require mandatory genetic confirmation of the mutation [7,15]. As a result, thousands of patients in developing countries remain completely cut off from access to modern treatments and clinical trials due to the lack of a genetic profile [5,7].

### 8.2. The High Cost of Innovation

The financial challenge extends beyond the cost of the DNA test itself and includes expenses for bioinformatics processing, variant-of-unknown significance (VUS) classification, and data storage [1,6]. At the same time, new gene and cell therapies come with high costs that strain even stable health insurance systems. Without international policies to support and facilitate technology transfer, there is a risk of reinforcing a bipolar model: a world in which “genetic code correction” is accessible only to a select few, while the rest of the patients remain stuck in the era of palliative care [1,7,8].

## 9. Conclusions and Future Prospects: The Skin as a Mirror of the Genome

The clinical management of epidermolysis bullosa (EB) is undergoing an unprecedented paradigm shift—from descriptive dermatology to precision genomic medicine. Five key pillars outline future developments in this field:

- Non-invasive diagnosis: NGS is finally replacing invasive skin biopsy. The future lies in early screening via oral swabs or prenatal diagnosis, which allows for early diagnosis and the preparation of a treatment plan even before birth.
- From ‘Type’ to ‘Mutation’: Disease classification is no longer merely descriptive (phenotypic) but is also functional (mutation specific). Every patient should have their own ‘genetic passport’, which determines whether they are suitable for allele-specific silencing, exon skipping, read-through therapy or genome editing.
- Overcoming Genetic Uncertainty (VUS): Decoding variants of unknown significance will rely on artificial intelligence (AI) and functional genomics to assess the biophysical effect of mutations on skin stability.
- Synchronisation between genetics and epigenetics: Understanding gene-modifying factors and the microenvironment will enable us to develop synergistic supportive therapies that ‘facilitate’ the action of the primary treatment and explain the clinical diversity observed among patients with identical underlying mutations.
- Democratisation of Technology: The major challenge facing the global community is to make genetic diagnostics and gene therapies economically accessible. The aim is for advances in molecular medicine to reach every ‘butterfly child’, regardless of their geographical location.

Molecular genetics has transformed epidermolysis bullosa from a dermatological tragedy into a triumph of genetic engineering [1,4,7]. The integration of NGS into clinical practice provides the precision that is fundamental to the successful application of new biological and gene therapies [5,15,18]. We are at the dawn of an era in which ‘butterfly children’ will not simply be bandaged, but their genetic code will be rewritten [9,15,18]. The path from decoding the code to correcting it has already been travelled. Now the most important step lies ahead: transforming these high-tech discoveries into accessible, everyday salvation for ‘butterfly’ children.

## Figures and Tables

**Table 1:** Types of EB.

EB Type	Level of Tissue Separation	Main Genes Involved
EB simplex (EBS)	Intraepidermal	*KRT5* (Keratin 5), *KRT14* (Keratin 14), *PLEC* (Plectin), *KLHL24* (Kelch Like Family Member 24)
Junctional EB (JEB)	Lamina lucida	*LAMA3* (Laminin Subunit Alpha 3), *LAMB3* (Laminin Subunit Beta 3), *LAMC2* (Laminin Subunit Gamma 2), *COL17A1* (Collagen Type XVII Alpha 1 Chain)
Dystrophic EB (DEB)	Sub-lamina densa	*COL7A1* (Collagen Type VII Alpha 1 Chain)
Kindler EB (KEB)	Mixed levels	*FERMT1* (Fermitin Family Member 1)

## Data Availability

No new data were created or analyzed in this study. Data sharing is not applicable to this article.

## Abbreviations

The following abbreviations are used in this manuscript:

AI	Artificial Intelligence
APOBEC	Apolipoprotein B mRNA-Editing Enzyme Catalytic Polypeptide-like
ASC	Adipose-derived Stem Cells
ASO	Antisense Oligonucleotide
B-VEC	Beremagene geperpavec (topical, replicationdefective herpes simplex virus type 1-based gene therapy)
CRISPR-Cas9	Clustered Regularly Interspaced Short Palindromic Repeats
cSCC	Cutaneous Squamous Cell Carcinoma
ctDNA	Circulating Tumor DNA
DDEB	Dominant Dystrophic Epidermolysis Bullosa
DEB	Dystrophic Epidermolysis Bullosa
EB	Epidermolysis bullosa
EBS	Epidermolysis bullosa simplex
EBS-MD	Epidermolysis Bullosa Simplex with Muscular Dystrophy
EBS-PA	Epidermolysis Bullosa Simplex with Pyloric Atresia
EGFR	Epidermal Growth Factor Receptor
EMT	Epithelial–Mesenchymal Transition
FDA	Food and Drug Administration
HSV-1	Herpes Simplex Virus Type 1
IFN-γ	Interferon-gamma
IFM	Immunofluorescence mapping
IL	Interleukin
iPSC	Induced Pluripotent Stem Cell
JEB	Junctional Epidermolysis Bullosa
KEB	Kindler Epidermolysis Bullosa
LNPs	lipid Nanoparticles
miRNAs	microRNAs
MMP	Matrix Metalloproteinase
MSC	Mesenchymal Stem Cell
nCATS	Nanopore Cas9-Targeted Sequencing
NGS	Next-generation sequencing
NMD	Nonsense-Mediated Decay
PCR	Polymerase Chain Reaction
PD-1	Programmed Cell Death Protein 1
PLK1	Polo-like Kinase 1
PTC	Premature Termination Codon
PTM	Pre-mRNA Trans-splicing Molecule
RDEB	Recessive Dystrophic Epidermolysis Bullosa
RM	Revertant Mosaicism
RNA-seq	RNA Sequencing
RTM	RNA Trans-splicing Molecule
SMaRT	Spliceosome-Mediated RNA Trans-splicing
SNP	Single Nucleotide Polymorphism
TGF-β	Transforming Growth Factor-beta
TNF-α	Tumor Necrosis Factor alpha
TNF-β	Tumor Necrosis Factor beta
TREx	Targeted RNA Sequencing
UC tissue-MSCs	Umbilical Cord tissue-derived Mesenchymal Stem Cells
VUS	Variants of unknown significance
WES	Whole Exome Sequencing
WGS	Whole Genome Sequencing
COL17A1	Collagen type XVII alpha 1 chain gene
COL7A1	Collagen type VII alpha 1 chain gene
FERMT1	Fermitin Family Member 1 gene
HRAS	HRas Proto-Oncogene, GTPase gene
KLHL24	Kelch-Like Family Member 24 gene
KRT14	Keratin 14 gene
KRT5	Keratin 5 gene
LAMA3	Laminin Subunit Alpha 3 gene
LAMB3	Laminin Subunit Beta 3 gene
LAMC2	Laminin Subunit Gamma 2 gene
MMP1	Matrix Metallopeptidase 1 gene
NOTCH1	Notch Receptor 1 gene
PLEC	Plectin gene
TP53	Tumor Protein P53 gene

## References

[1] Bardhan A., Bruckner-Tuderman L., Chapple I.L., Fine J.D., Harper N., Has C., Magin T.M., Marinkovich M.P., Marshall J.F., McGrath J.A. (2020). Epidermolysis bullosa. Nat. Rev. Dis. Primers.

[2] Has C., Bauer J.W., Bodemer C., Bolling M.C., Bruckner-Tuderman L., Diem A., Fine J.D., Heagerty A., Hovnanian A., Marinkovich M.P. (2020). Consensus reclassification of inherited epidermolysis bullosa and other disorders with skin fragility. Br. J. Dermatol..

[3] Fine J.-D., Bruckner-Tuderman L., Eady R.A., Bauer E.A., Bauer J.W., Has C., Heagerty A., Hintner H., Hovnanian A., Jonkman M.F. (2014). Inherited epidermolysis bullosa: Updated recommendations on diagnosis and classification. J. Am. Acad. Dermatol..

[4] McGrath J.A. (2017). The molecular revolution in cutaneous biology: Era of molecular diagnostics for inherited skin diseases. J. Investig. Dermatol..

[5] Baardman R., Lemmink H.H., Yenamandra V.K., Commandeur-Jan S.Z., Viel M., Kooi K.A., Diercks G.F.H., Meijer R., van Geel M., Scheffer H. (2025). Evolution of genome diagnostics in epidermolysis bullosa: Unveiling the power of next-generation sequencing. J. Eur. Acad. Dermatol. Venereol..

[6] King A.D., Deirawan H., Klein P.A., Dasgeb B., Dumur C.I., Mehregan D.R. (2023). Next-generation sequencing in dermatology. Front. Med..

[7] Danescu S., Negrutiu M., Has C. (2024). Treatment of epidermolysis bullosa and future directions: A review. Dermatol. Ther..

[8] Welponer T., Prodinger C., Pinon-Hofbauer J., Hintersteininger A., Breitenbach-Koller H., Bauer J.W., Laimer M. (2021). Clinical perspectives of gene-targeted therapies for epidermolysis bullosa. Dermatol. Ther..

[9] Kocher T., Koller U. (2021). Advances in gene editing strategies for epidermolysis bullosa. Prog. Mol. Biol. Transl. Sci..

[10] Mirghasemi N. (2025). CRISPR-based gene editing therapy for epidermolysis bullosa simplex: Molecular targets and therapeutic strategies. Am. J. Stud. Res..

[11] Vermeer F.C., Bremer J., Sietsma R.J., Sandilands A., Hickerson R.P., Bolling M.C., Pasmooij A.M., Lemmink H.H., Swertz M.A., Knoers N.V. (2021). Therapeutic prospects of exon skipping for epidermolysis bullosa. Int. J. Mol. Sci..

[12] Nyström A., Bruckner-Tuderman L., Kiritsi D. (2021). Dystrophic epidermolysis bullosa: Secondary disease mechanisms and disease modifiers. Front. Genet..

[13] Richards S., Aziz N., Bale S., Bick D., Das S., Gastier-Foster J., Grody W.W., Hegde M., Lyon E., Spector E. (2015). Standards and guidelines for the interpretation of sequence variants: A joint consensus recommendation of the American College of Medical Genetics and Genomics and the Association for Molecular Pathology. Genet. Med..

[14] Rehm H.L., Berg J.S., Brooks L.D., Bustamante C.D., Evans J.P., Landrum M.J., Ledbetter D.H., Maglott D.R., Martin C.L., Nussbaum R.L. (2015). ClinGen—The Clinical Genome Resource. N. Engl. J. Med..

[15] Bischof J., Hierl M., Koller U. (2024). Emerging gene therapeutics for epidermolysis bullosa under development. Int. J. Mol. Sci..

[16] Koller U., Bauer J.W. (2024). Emerging DNA & RNA editing strategies for the treatment of epidermolysis bullosa. J. Dermatol. Treat..

[17] Wang X., Wang X., Li Y., A S., Qiu B., Bushmalyova A., He Z., Wang W., Lara-Sáez I. (2023). CRISPR-Cas9-based non-viral gene editing therapy for topical treatment of recessive dystrophic epidermolysis bullosa. Mol. Ther. Methods Clin. Dev..

[18] Guide S.V., Gonzalez M.E., Bağcı I.S., Agostini B., Chen H., Feeney G., Steimer M., Kapadia B., Sridhar K., Sanchez L.Q. (2023). Trial of dystrophic epidermolysis bullosa gene therapy (B-VEC). N. Engl. J. Med..

[19] Koller U. (2023). Gene therapy shine the spotlight on epidermolysis bullosa, bringing hope to patients. Mol. Ther..

[20] Cattaneo C., Enzo E., De Rosa L., Sercia L., Consiglio F., Forcato M., Bicciato S., Paiardini A., Basso G., Tagliafico E. (2024). Allele-specific CRISPR-Cas9 editing of dominant epidermolysis bullosa simplex in human epidermal stem cells. Mol. Ther..

[21] Zimmer D., Li V., Mukarram M., Frasier K., Herrick G., Vinagolu-Baur J., Kakarla S., Coleman M. (2024). CRISPR-Cas9 Gene Editing in Pediatric Epidermolysis Bullosa. Am. J. Clin. Med. Res..

[22] Koutsoukos S.A., Bilousova G. (2024). Highlights of gene and cell therapy for epidermolysis bullosa and ichthyosis. Dermatol. Ther..

[23] Jacków J., Guo Z., Hansen C., Abaci H.E., Doucet Y.S., Shin J.U., Hayashi R., DeLorenzo D., Kabata Y., Shinkuma S. (2019). CRISPR/Cas9-based targeted genome editing for correction of recessive dystrophic epidermolysis bullosa using iPS cells. Proc. Natl. Acad. Sci. USA.

[24] Cho R.J., Alexandrov L.B., Breems N.Y.D., Atanasova V.S., Farshchian M., Purdom E., Nguyen T.N., Coarfa C., Rajapakshe K., Prisco M. (2018). APOBEC mutation drives early-onset squamous cell carcinomas in recessive dystrophic epidermolysis bullosa. Sci. Transl. Med..

[25] Badowski C., Tan T.S., Aliev T., Trudil D., Larina M., Argentova V., Firdaus M.J., Benny P., Woo V.S., Lane E.B. (2022). Detrimental effects of IFN-γ on an epidermolysis bullosa simplex cell model and protection by a humanized anti-IFN-γ monoclonal antibody. JID Innov..

[26] Meyer-Mueller C., Osborn M.J., Tolar J., Boull C., Ebens C.L. (2022). Revertant mosaicism in epidermolysis bullosa. Biomedicines.

[27] Natsuga K., Furuta Y., Takashima S., Nohara T., Kosumi H., Mai Y., Higashi H., Ujiie H. (2022). Detection of revertant mosaicism in epidermolysis bullosa through Cas9-targeted long-read sequencing. Hum. Mutat..

[28] Twaroski K., Eide C., Riddle M., Xia L., Lees C., Chen W., Mathews W., Keene D., McGrath J., Tolar J. (2019). Revertant mosaic fibroblasts in recessive dystrophic epidermolysis bullosa. Br. J. Dermatol..

